# Recombinant Human Brain Natriuretic Peptide Attenuates Myocardial Ischemia-Reperfusion Injury by Inhibiting CD4^+^ T Cell Proliferation via PI3K/AKT/mTOR Pathway Activation

**DOI:** 10.1155/2020/1389312

**Published:** 2020-06-12

**Authors:** Kun-Peng Li, Hai-Yan Zhang, Xiao-Dong Xu, Ming-Yang, Tie-Jun Li, Shu-Tian Song

**Affiliations:** ^1^Department of Cardiothoracic Surgery, The Cangzhou Central Hospital, Hebei Province 061001, China; ^2^Department of Nursing, Cangzhou Medical College, Hebei Province 061001, China; ^3^Department of Medicine, Cangzhou Medical College, Hebei Province 061001, China; ^4^Department of Anesthesiology, Cangzhou Central Hospital, Hebei Province 061001, China

## Abstract

Inflammation plays a major role in the development of myocardial ischemia-reperfusion (IR) injury. Recombinant human brain natriuretic peptide (rhBNP), a man-made version of a peptide that is elevated in heart failure, exhibits anti-inflammatory effects in various tissues. However, its role in myocardial IR injury remains unclear. In this study, we demonstrate that treatment with rhBNP provided protection for mice against myocardial IR injury as manifested by reduced infarct size and well-preserved myocardial, attenuated inflammatory infiltration and CD4^+^ T cell proliferation function, and inhibited expression of proinflammatory related genes. Furthermore, mechanistic studies revealed that rhBNP inhibited Jurkat T proliferation by promoting PI3K/AKT/mTOR phosphorylation. Collectively, our data suggest that the administration of rhBNP during IR injury could expand our understanding of the cardioprotective effects of rhBNP.

## 1. Introduction

Myocardial ischemia-reperfusion (IR) injury is a major contributor to the morbidity and mortality of coronary artery disease. IR injury causes progressive necrotic and apoptotic cell death, which compromises cardiac contractility and electrophysiological performance [1]. A critical problem remaining in clinical practice is how to balance the reestablishment of the blood supply to ischemic myocardial tissue against the need to minimize or avoid IR injury.

The PI3K/AKT/mTOR signaling pathway is essential for control of CD4^+^ T cell development, function, and stability in mammalian cells [2]. There is overwhelming evidence that IR affects immune homeostasis by causing changes in endothelial, tubular epithelial, and renal parenchymal cells, as well as leukocytes [3, 4]. The main innate immune response to IR injury involves the activation and accumulation of T cells in the postischemic heart [5–7]. Depletion of CD4^+^ T cells in animal models is sufficient to reduce myocardial IR injury [8]. Additionally, conventional CD4^+^ T cells, including Th1 and Th17, play a major role in the development of IR injury [9]. In one study, elimination of T cells by use of lymphocyte-deficient RAG1 knockout (KO) mice protected against IR injury [10]. In another study, mice depleted of CD4^+^ T cells, but not of CD8^+^ T cells, had significantly smaller infarcts compared with wild-type mice [7]. Therefore, an improved understanding of the underlying molecular mechanisms would be instrumental for the development of CD4^+^ T cell-based strategies to protect against myocardial IR injury.

Brain natriuretic peptide (also known as B-type natriuretic peptide or BNP) is the predominant natriuretic peptide in mammalian myocardium. As a cardiac hormone produced mainly by ventricular myocytes, BNP has served as a biomarker for heart failure [11, 12]. Measurement of serum BNP concentrations has high sensitivity and specificity for heart failure diagnosis [13]. Several studies have shown that BNP may reduce myocardial IR injury [14–16]. Moreover, a recent study demonstrated that BNP functions as an anti-inflammatory that protects the heart from multiple complications [14]. Recombinant human BNP (rhBNP) is a man-made peptide, developed through gene engineering, that is widely used to manage acute uncompensated congestive heart failure in patients [12]. Recently, rhBNP has also been administered intravenously to guide fluid therapy and predict outcomes of acute illness including IR injury in critical care units [17]. However, the effect of rhBNP on myocardial IR injury and the underlying mechanism for this remains unclear. Thus, we aimed at determining whether rhBNP exerts its protective effect in IR injury by regulating CD4^+^ T cell homeostasis and to ascertain the mechanism involved.

## 2. Materials and Methods

### 2.1. Reagents

rhBNP was purchased from Nuodikang Biological Pharmaceutical Co. Ltd. (Chengdu, China). Antibodies were obtained from Cell Signaling Technology (Danvers, USA).

### 2.2. Cell Line and Animals

Adult C57BL/6 mice (aged 8-10 weeks, weighing 20-25 g) were purchased from Shanghai SipprBK Lab Animal Co. Ltd. (Shanghai, China) and housed in an air-conditioned room at 23 ± 2°C and with a 12 h light/dark cycle. Water and food were available *ad libitum*. All experiments were conducted in accordance with the National Institutes of Health Guidelines for the Care and Use of Laboratory Animals. This experiment was approved by the medical ethics committee of the Cangzhou central hospital of Hebei province.

Jurkat T cells (a human T cell line) were obtained from the Shanghai Institute of Cell Biology, Chinese Academy of Sciences (Shanghai, China) and were cultured in RPMI-1640 containing 10% (*v*/*v*) FBS (Hyclone, USA), 50 U/mL penicillin, and 50 *μ*g/mL streptomycin (complete medium) in a humidified chamber containing 5% CO_2_ at 37°C.

### 2.3. Groups and Treatments

Sixty mice were divided into three groups (*n* = 6 per group): sham + phosphate-buffered saline (PBS), IR + PBS, and IR + rhBNP, consistent with the prior publication [17, 18]. The mice were received an intraperitoneal injection of 0.035 mg of rhBNP or isopyknic PBS every other day for a total of three times after performing the IR surgery.

Jurkat T cells were divided into two groups: control and rhBNP (0.1 *μ*M), as described previously [19].

### 2.4. Model Establishment

Mice were fasted for 12 h preoperatively and then anesthetized with 1-2% isoflurane before inducing IR as described elsewhere [6]. Briefly, myocardial ischemia was induced temporarily by placing a silk suture (6–0) slipknot around the left anterior descending coronary artery via a left thoracic incision. The slipknot was released 40 min after myocardial infarction and then the myocardium was reperfused. At 24 h after the surgery, blood samples were collected and centrifuged (3,500 × g) for 15 min before collecting the supernatant serum for storage at -80°C.

### 2.5. Infarct Size Measurement

Myocardial infarct size was measured with 2,3,5-triphenyltetrazolium chloride (TTC) staining. Mice were sacrificed by cervical dislocation, and the heart tissue harvested and stored immediately at –20°C for 40 min. Each heart was sliced perpendicular to the vertical axis into 5 uniform sections. The 2 mm thick sections were incubated in 1% TTC solution at 37°C for 30 min in the dark and then immersed in 4% paraformaldehyde overnight. The infarct area was determined by computerized planimetry and the area percentage calculated.

### 2.6. Cardiac Marker Enzymes and Cardiac Troponin T

Blood was collected 180 min after reperfusion, centrifuged (3000 rpm) for 10 min at 4°C, and the serum was drawn into microtubes for storage at -20°C until assay. Plasma creatine kinase (CK, Zhongshengbeikong, Beijing, China), lactate dehydrogenase (LDH, Zhongshengbeikong, Beijing, China), and troponin T (TnT, Roche Diagnostics, New York, USA) concentrations were determined as per the instructions provided with the purchased kits.

### 2.7. Histopathology

After IR, paraffin-embedded tissue samples were sectioned serially into 3-5 *μ*m thick slices, dewaxed, and dehydrated before staining with hematoxylin-eosin (HE) and observing under a light microscope (Leica Microsystems, Germany).

### 2.8. Flow Cytometric Analysis

For isolation of single cells from the mouse heart, we used a previously described protocol [20]. Briefly, mice were intracardiac perfused with 50 mL of ice-cold Hank's balanced salt solution (HBSS) with heparin to exclude blood cells. Left ventricular was dissected, minced with fine scissors, and digested with collagenase type and protease type XIV (Sigma Aldrich, USA) and filtered through 40 *μ*m cell strainers (Merck Millipore, USA).

Cells were labeled for surface expression of the following anti-mouse Abs: anti-CD3e (17A2), anti-CD4 (GK1.5 and RM4-5), anti-CD8 (53-6.7), anti-CD25 (PC61), and anti-CD69 (H1.2F3). For intracellular analysis, cells were permeabilized and stained with anti-Ki67 (35/ki-67). All Abs were purchased from BD Bioscience (USA) or eBioscience (USA). FACS Aria I (BD Biosciences, USA) was used to collect the data, and FlowJo software (Tree Star) was used for analysis.

### 2.9. Western Blot

Western blot assay was conducted as described previously [21]. In brief, cells were lysed with RIPA lysis buffer containing 1 mmol/L cocktail. Homogenates were then prepared and centrifuged (12,000 g) at 4°C for 10 min. Protein concentration was determined by the BCA method. Nonspecific binding was blocked with 5% skimmed milk for 1.5 h, and the membranes were incubated with p-AKT S473, total AKT and p-PI3K (BD Biosciences, USA) together with total PI3K, p-mTOR S2448 and total mTOR (Biolegend, USA), and p-P70 S6 kinase (p-P70S6K), P70 S6 kinase (P70S6K) and GAPDH (Santa Cruz Biotechnology, USA) at 4°C overnight and in the secondary antibody at room temperature for 1 h. The signals were determined using the Amersham prime ECL Plus detection system (Pittsburgh, PA).

### 2.10. Quantitative Real-Time PCR

RNA was extracted using TRIzol (Invitrogen, USA), and first-strand cDNA synthesis was performed using Advantage RT-for-PCR (BD Biosciences). For conventional reverse transcription polymerase chain reaction (RT-PCR), cDNA was amplified using Taq DNA polymerase (Eppendorf, Hamburg, Germany). Primers were designed to span exon-exon junctions. Comparable quantities of cDNA were ensured via amplification of *β*-actin (forward, 5′-CAGCCTTCCTTCTTGGGTATGG-3′ and reverse, 5′-CGCAGCTCAGTAACAGTCCG-3′). For amplification of other mRNA, we used the following primers, interleukin- (IL-) 1*β* (forward, 5′-ATGCCTCGTGCTGTCTGACC-3′ and reverse, 5′-CCATCTTTAGGAAGACACGGGTT-3′), tumor necrosis factor- (TNF-) *α* (forward, 5′-AGCGGCTGACTGAACTCA GATTGTAG-3′ and reverse, 5′-GTCACAGTTTTCAGCTGTATAGGG-3′), IL-10 (forward, 5′-AGTGGAGCAGGTGAAGAGTG-3′, reverse, 5′-TTCGGAGAGAGGTACAAACG-3′), transforming growth factor- (TGF-) *β* (forward, 5′-GCTACCATGCCAACTTCTGT3′, reverse, 5′-CGTAGTAGACGATGGGCAGT-3′).

### 2.11. Statistical Analyses

All data are presented as the mean ± standard deviation (SD). For analysis of differences between two groups, unpaired Student's *t* test was performed. For multiple groups, one-way ANOVA was performed, followed by the Bonferroni post-hoc test. GraphPad Prism 5.0 (La Jolla, USA) software was used to calculate the significance between groups. A value of *p* < 0.01 was considered statistically significant.

## 3. Results

### 3.1. Treatment with rhBNP Protects the Heart from IR Injury

**Figure 1:**
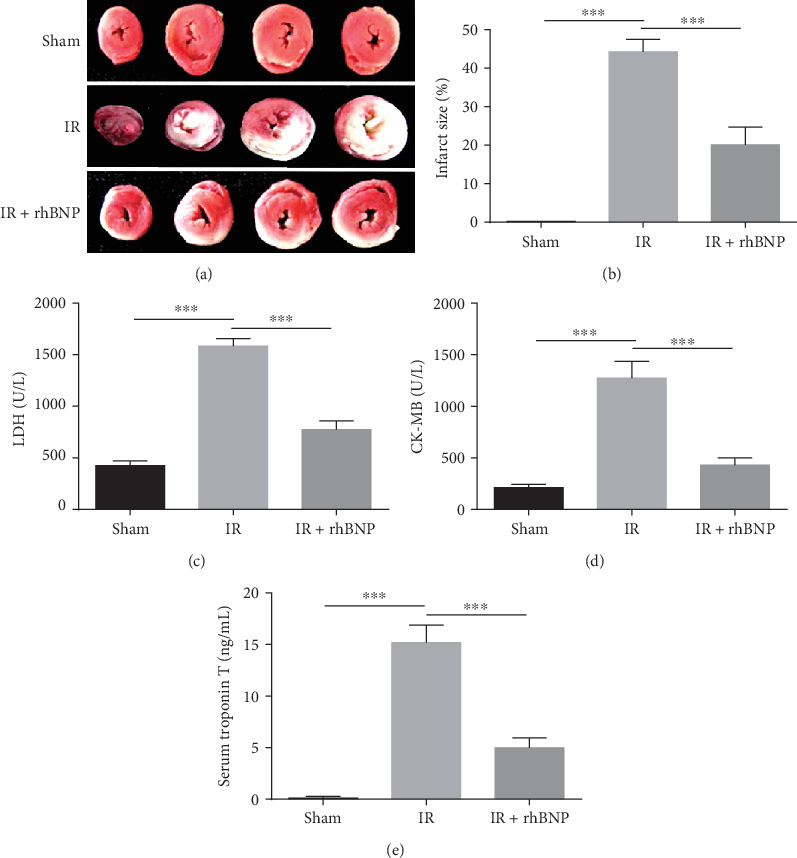
Treatment with rhBNP protects the heart from IR injury. (a, b) IR model mice with/without rhBNP pretreatment were assessed for infarct size by TTC assay: red stain represents the viable area, and white stain represents the infarcted region. Serum concentrations of (c) LDH, (d) CK, and (e) TnT in sham, IR, and IR+rhBNP groups. Sham: sham-operated group; IR: model group; IR+rhBNP: model mice treated with rhBNP (0.035 mg/kg). LDH: lactate dehydrogenase; CK: creatine kinase; TnT: troponin T; *n* = 5 per group, data are expressed as mean ± SD, ^∗∗∗^*p* < 0.01.

To confirm the effects of rhBNP on myocardial IR injury, we used TCC staining to evaluate the infarction area.  Figure 1(a) presented the photographs of stained cardiac tissue in each group. And the infarction area in the IR group was 44.26% greater than in the sham group, and treatment with rhBNP significantly reduced the infarction to 20.12% (Figure 1(b)). This suggests that myocardial damage was significantly reduced by rhBNP. Furthermore, mean serum concentrations of the cardiac enzymes, LDH and CK, in the IR group were elevated at 1582.00 ± 74.59 U/L (Figure 1(c)) and 1273.17 ± 162.67 U/L (Figure 1(d)), respectively, compared with 773.50 ± 85.30 U/L (Figure 1(c)) and 431.00 ± 69.65 (Figure 1(d)), respectively, in the sham group. In contrast, the rhBNP group had significantly lower LDH and CK concentrations at 836.50 ± 45.33 and 487.83 ± 40.23 U/L, respectively. Moreover, cardiac TnT production was lower in the rhBNP-treated group at 4.99 ± 0.93 ng/mL compared with 15.18 ± 1.69 ng/mL in the IR group (*p* < 0.01) and 0.10 ± 0.20 ng/mL in the sham group (Figure 1(e)). These findings suggest a cardioprotective effect of rhBNP.

### 3.2. rhBNP Reduces the Number and Proportion of T Cells

**Figure 2:**
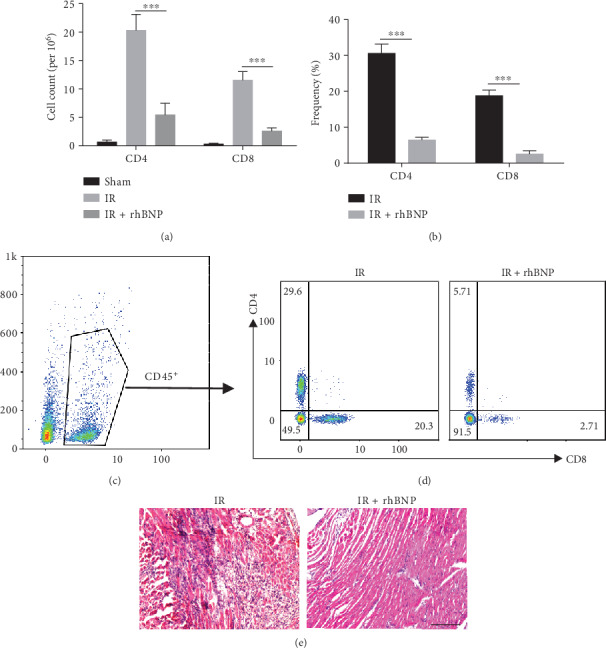
Effects of rhBNP on myocardial T cells. (a) Absolute cell numbers and (b) percentages of CD4^+^ and CD8^+^ T cells. (c) Representative dot plots of the CD45^+^, CD4^+^, or CD45^+^CD8^+^ subset in the myocardium of the IR and IR+rhBNP groups. (d) HE staining of heart tissue. Original magnification (200x). (e) Sham: sham-operated group; IR: model group; IR+rhBNP: model mice treated with rhBNP (0.035 mg/kg); *n* = 5 per group, data are expressed as mean ± SD, ^∗∗∗^*p* < 0.01.

We evaluated T cell development in mice with myocardial IR injury in the presence and absence of rhBNP treatment. After IR injury, large numbers of CD4^+^ and CD8^+^ T cells assembled in the heart, but the number (Figure 2(a)) and proportion (Figures 2(b)–2(d)) of these cells decreased slightly with rhBNP treatment. Consistently, the HE staining assay showed that inflammatory infiltration was substantially reduced in IR mice treated with rhBNP (Figure 2(e)). These data show that rhBNP could reduce T cell-related inflammatory infiltration after IR injury.

### 3.3. rhBNP Limits T Cells Proliferation but Not Activation

**Figure 3:**
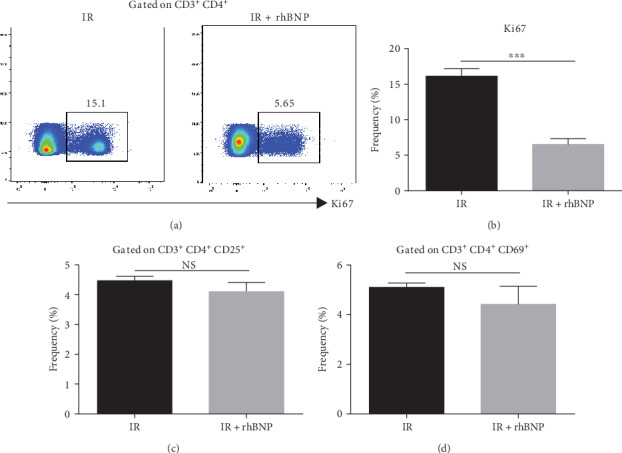
Effects of rhBNP on T cell proliferation and activation. Cell (a, b) proliferation and (c, d) activation was determined by flow cytometry after labelling CD3^+^ and CD4^+^ antibodies. IR: model group; IR+rhBNP: model mice treated with rhBNP (0.035 mg/kg). *n* = 3 per group, data are expressed as mean ± SD, ^∗∗∗^*p* < 0.01, NS: no significant difference.

To address the functional impact of rhBNP on T cells, we assessed T cell proliferation by FACS. CD3^+^ cells in mice with IR treated with rhBNP had significantly induced T cell proliferation compared with the IR group that did not receive rhBNP (Figures 3(a) and 3(b)). On the other hand, the T cell activation markers, CD25 and CD69, showed no significant difference with rhBNP treatment (Figures 3(c) and 3(d)). Collectively, these data show that rhBNP inhibited T cell proliferation, but played no role in T cell activation.

### 3.4. rhBNP Downregulates Inflammatory Cytokine Expression in Myocardial Tissue

Myocardial IR is usually associated with severe inflammatory reactions and expression of inflammatory cytokines such as IL-1*β* and TNF-*α* while anti-inflammatory cytokines such as TGF*β* and IL-10 are suppressed [7]. We used RT-PCR to determine the levels of inflammatory and anti-inflammatory cytokines in myocardial tissue.

**Figure 4:**
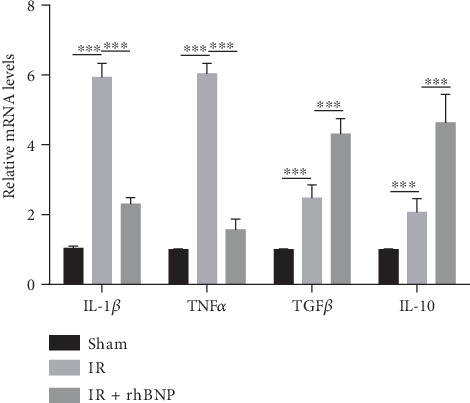
rhBNP regulates inflammatory cytokine expression. Quantitative analysis of IL-1*β*, TNF*α*, TGF*β*, and IL-10 mRNA expression in the myocardium from sham, IR, and IR+rhBNP groups. *n* = 3 per group. Sham: sham-operated group; IR: model group; IR+rhBNP: model mice treated with rhBNP (0.035 mg/kg). Data are expressed as mean ± SD. ^∗∗∗^*p* < 0.01.

Figure 4 shows that the expression of IL-1*β* and TNF-*α* was higher in the IR group compared with the sham group, while the expression of TGF*β* and IL-10 was lower. Nevertheless, rhBNP effectively inhibited the increase in inflammatory factor levels and reversed the decrease in anti-inflammatory factor levels compared with the IR group.

### 3.5. rhBNP Inhibits Jurkat T Cells Proliferation

**Figure 5:**
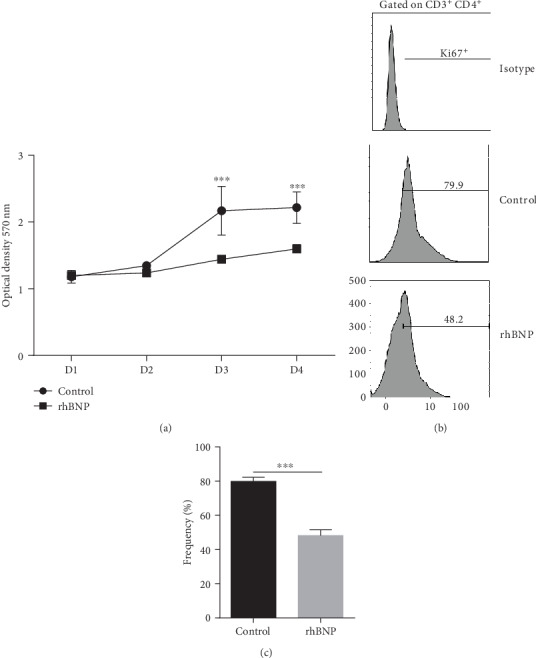
Effects of rhBNP on Jurkat T cell proliferation. (a) Cell viability was detected by CCK-8 assay. (b, c) Cell proliferation was determined by flow cytometry after labelling CD3^+^ and CD4^+^ antibody. Control: cells treated with equivalent PBS. rhBNP: cells treated with rhBNP (0.1 *μ*M). *n* = 3 per group, data are expressed as mean ± SD. ^∗∗∗^*p* < 0.01.

To investigate the effect of rhBNP metabolites on T cells, Jurkat cells were treated with rhBNP and activated using human anti-CD3/CD28 beads. CCK-8 assay results showed that the optical density (OD) value of cells treated with rhBNP was significantly lower than that of cells in the control group (p < 0.05 on days 3 and 4, Figure 5(a)). Furthermore, the Ki67 labeling cell proliferation assay showed that Jurkat T cell proliferation decreased by about 40% with rhBNP treatment. These findings suggested that rhBNP also inhibited the proliferation of Jurkat T cells.

### 3.6. rhBNP Decreases Phosphorylation of the PI3K/AKT Pathway in Jurkat T Cells

**Figure 6:**
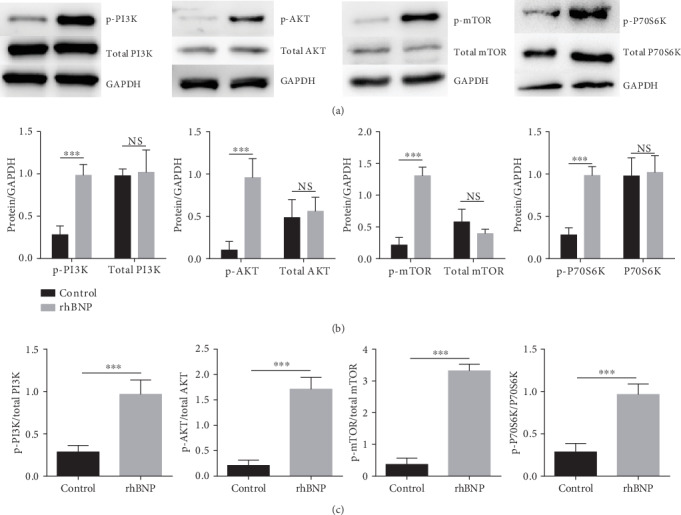
Effects of rhBNP on the PI3K/AKT/mTOR pathway in Jurkat T cell. (a) Western blot analysis for total and phosphorylated PI3K, AKT, mTOR, and P70S6K in cells. (b) Comparison of the expression level of total and phosphorylated PI3K, AKT, mTOR, and P70S6K. (c) Comparison of the activation level of these proteins in Jurkat T cells with/without rhBNP (0.1 *μ*M) treatment. *n* = 3 per group, data are expressed as mean ± SD. ^∗∗∗^*p* < 0.01.

To gain insight into the mechanisms of rhBNP inhibition of T cell proliferation, we examined the activities of PI3K (p-PI3K), AKT (p-AKT), mTOR (p-mTOR), and P70S6K (p-P70S6K) by western blot analysis (Figure 6). We showed that rhBNP treatment promoted phosphorylation of PI3K, AKT, and mTOR compared with the control group (*p* < 0.05). However, no differences were detected in total protein (total PI3K, total AKT, total mTOR, and total P70S6K, Figure 6(b)).

## 4. Discussion

The therapeutic effect of rhBNP in the clinical management of cardiac diseases is well recognized, as is its ability to exert anti-inflammatory activity in various organs [22, 23]. There is clearly a beneficial effect of rhBNP in myocardial IR injury, but the underlying mechanism for this remains unclear. The adaptive immune response, especially that which involves CD4^+^ T cells [9], is important to the wound-healing process [8]. Recently, there is robust evidence from mouse models for an important role for CD4^+^ T cells in ischemia-reperfusion [4, 10]. Several studies have used CD4^+^ T cell-deficient mice (CD4^+^ KO and RAG1 KO) and demonstrated an emerging contribution of CD4^+^ T cells in IR injury and infarct healing [8, 10]. The establishment of the mechanism underlying the influence of rhBNP on CD4^+^ T cells in IR injury has significant clinical implications. Our results indicate that rhBNP inhibits T cell proliferation in the IR area by promoting phosphorylation of the PI3K/AKT/mTOR pathway.

We studied the protective effect of rhBNP on IR injury in mice and found that rhBNP (0.035 mg/kg) preconditioning reduced infarct size (Figures 1(a) and 1(b)) along with myocardial kinase (LDH and CK, Figures 1(c) and 1(d)) and TnT (Figure 1(e)) levels, which was consistent with previous studies. To explore the anti-inflammatory effects of rhBNP on CD4^+^ T cells, we used FACS to analyze the number and proportion of CD4^+^ and CD8^+^ T cells in diseased cardiac tissue and showed that rhBNP treatment reduced both cell types in the heart (Figures 2(a)–2(d)). The HE staining assay also showed that rhBNP reduced inflammatory infiltration (Figure 2(e)).

We evaluated the effects of rhBNP on CD4^+^ T cells and showed that the expression of ki67 in CD3^+^ CD4^+^ T cells decreased (Figures 3(a) and 3(b)), indicating that rhBNP inhibited CD4^+^ T cell proliferation. CD4^+^ T cell activation was not affected, as tested through the determination of the expression of CD25 and CD69 (Figures 3(c) and 3(d)), two markers of T cell activation. IL-1*β* and TNF*α* are two important proinflammatory cytokines [24, 25], whereas TGF*β* and IL-10 are anti-inflammatory [26]. Moreover, we found that the mRNA levels of IL-1*β* and TNF*α* increased after IR but decreased after treatment with rhBNP. In contrast, mRNA levels of TGF*β* and IL-10 increased after treatment with rhBNP. Consistently, a recent study showed that, during IR, BNP inhibited the expression of inflammation-related mRNA, including TNF-*α* and IL-6 [8]. Totally, our i*n vitro* experiments demonstrated that rhBNP can directly affect T cells.

To address the mechanisms relevant to rhBNP in T cells, we treated Jurkat T cells, a widely used leukaemic T cell line, with rhBNP to explore whether rhBNP had the same effect on proliferation. The MTT assay showed that cell growth rate was significantly inhibited after rhBNP (0.1 *μ*M) treatment (Figure 5(a)). To confirm these results, we further examined ki67 cell expression by FACS (Figure 5(b)) and showed that the addition of rhBNP significantly inhibited T cell proliferation.

The PI3K/AKT/mTOR signaling pathway is involved in many aspects of cell growth, cellular activation, and inflammatory response under pathological conditions [27]. Activation of PI3K/AKT-dependent signaling prevents cardiomyopathy and apoptosis and protects against myocardial IR injury [28]. We found that rhBNP promoted phosphorylation of PI3K and its downstream components (AKT, mTOR, and P70S6K), but not total protein expression (Figure 6).

In conclusion, we showed that rhBNP inhibited myocardial IR injury by inhibiting CD4^+^ T cell proliferation through the promotion of PI3K/AKT/mTOR pathway phosphorylation.

## 5. Conclusion

We have provided convincing evidence that pretreatment of mice with rhBNP protects against myocardial IR injury as manifested by the attenuated inflammatory infiltration and reduced CD4^+^ T cell proliferation with preserved myocardial function. Mechanistic studies revealed that rhBNP suppresses T cell proliferation by regulating PI3K/AKT/mTOR signaling. Together, this novel aspect of rhBNP action in myocardial IR injury expands our understanding of the cardioprotective effects of rhBNP.

## Data Availability

The data used to support the findings of this study are available from the corresponding author upon request.
